# Results of Re-vaccination

**Published:** 1853-10

**Authors:** 


*Results of Re-vaccination.*—Dr. Herapath, of Bristol, Eng., draws the following inferences from the results of 257 cases of re-vaccination in two public schools in that city with which he is professionally connected, one containing at the time of the experiment, 101 girls, and the other 158 boys. The ages of the patients all ranged between eight and seventeen and a half years:

1. That those cases re-vaccinated within seven years are not again susceptible to vaccine.

2. That vaccine, after the interval of from eight to seventeen years, does not prevent the reception of vaccine again, except in 22.174 per cent.

3. That the distinctness or imperfection of the vaccine cicatrix does not materially alter these results.

4. That variola does not prevent the formation of the vaccine vesicle, except in about 23.53 per cent.

5. That the recurrence of small-pox subsequently to vaccination, does not destroy the susceptibility of the human system again to receive the vaccine poison, except in about ten per cent.

6. That in all the previous cases, whenever the secondary vaccine vesicle assumed its perfect form, its subsequent history was the same as if the system had not previously labored under vaccine variola or varioloid.

7. It is probable that the protective influence of vaccination has diminished in consequence of repeated transmission of the vaccine matter through the human body.

8. It is desirable that re-vaccination should be extensively followed, as one means of giving additional protection to the masses.

9. That when possible, the stock of vaccine should be renewed by going back to the original source.—*Association Med. Jour.*, April 15, 1853, from *N. Y. Med. Times.*

